# Weather-Robust Foreign Object Detection on Transmission Lines via Physics-Driven Complex Wavelet Unrolling

**DOI:** 10.3390/s26102942

**Published:** 2026-05-08

**Authors:** Xiaoxiong Zhou, Junchi He, Cheng Cheng, Guangming Zhang

**Affiliations:** 1 College of Electrical Engineering and Control Science, Nanjing Tech University, Nanjing 211816, China; zhouxiaoxiong@njtech.edu.cn; 2 Distribution Network Operation and Maintenance Center, Guanyun County Electric Power Supply Company, State Grid Jiangsu Electric Power Co., Ltd., Lianyungang 222207, China; hejunchi1993@163.com (J.H.); chengc1993@njtech.edu.cn (C.C.)

**Keywords:** foreign object detection, adverse condition, two-dimensional dual-tree complex wavelet transform, physical priors

## Abstract

Foreign object detection during unmanned aerial vehicle (UAV) grid inspection suffers from severe visual degradation under adverse weather conditions, such as haze and heavy rain. Existing approaches often struggle to distinguish target textures from weather-induced noise, leading to critical performance drops. We propose the Physics-Prior Complex Wavelet Unrolling Decoupling Module (PCW-UDM) to enable highly robust detection in complex environments. By leveraging the 2D dual-tree complex wavelet transform (2D-DTCWT), our method decouples degraded features into low-frequency and multi-directional high-frequency sub-bands. To tackle haze, we design a Physics-Guided Low-Frequency Dehazing (PGLD) branch that physically inverses the atmospheric scattering process. To combat rain, we introduce the LISTA-Unrolled High-Frequency Deraining (LUHD) branch, which innovatively applies deep unrolled sparse optimisation to remove directional rain streaks without distorting the structural phase. A novel spatio-temporal cross-domain consistency loss further guarantees weather-invariant feature alignment. Extensive evaluations on synthesised adverse datasets and the real-world RTTS dataset prove that our PCW-UDM-equipped network fundamentally overcomes the semantic conflicts of traditional cascaded restoration–detection paradigms, achieving state-of-the-art detection precision and robustness against extreme weather conditions.

## 1. Introduction

With the rapid development of smart grids, not only has the coverage of power transmission lines expanded, but their operational safety has been directly linked to the stability of the power system. Foreign objects attached to power transmission lines (such as kites and birds’ nests) can easily cause short circuits and tripping, damage to equipment, or even localised grid failures; therefore, the timely and accurate detection of foreign objects on power transmission lines is of great significance [1,2,3]. Traditional inspection methods rely primarily on manual checks, which are not only labour-intensive and inefficient but are also constrained by factors such as inspection distance, viewing angles, and image quality, making it difficult to meet the demands of large-scale, routine inspections [4,5]. In recent years, with the development of unmanned aerial vehicle (UAV) platforms and computer vision technology, power transmission line inspections have gradually shifted from traditional manual methods to intelligent visual inspection methods [5,6,7,8]. Multi-fault insulator detection based on an improved. Visual inspection refers to a class of technologies that utilise image or video data to automatically achieve target recognition, localisation and classification through algorithms.

In the field of visual detection tasks, relevant intelligent algorithms have gradually evolved from early traditional methods reliant on manually defined features to end-to-end detection frameworks based on deep learning. Especially in the domain of object detection, deep learning methods have demonstrated significant advantages in feature extraction, multi-scale representation, and the recognition of small objects, providing effective support for automated detection in complex industrial settings. With regard to foreign object detection on power transmission lines, existing research has made significant progress in optimising backbone networks, lightweight design, and the incorporation of attention mechanisms, thereby effectively enhancing detection accuracy and model efficiency [4,9,10,11,12,13].

However, although existing methods have effectively improved the detection performance of foreign objects on power transmission lines, complex and adverse weather conditions pose even more severe challenges to drone inspections. Power transmission lines frequently traverse mountainous and high-altitude regions, which are often plagued by haze and torrential rain [14,15,16]. Under these adverse conditions, optical sensors suffer severe visual degradation, where atmospheric scattering caused by haze significantly reduces image contrast, whilst directional rain streaks obscure the geometric textures of transmitted components [17,18,19,20]. Models trained on datasets from clear weather conditions experience a rapid decline in detection performance when faced with adverse environments, rendering them unsuitable for reliable deployment in real-world scenarios. Existing research typically improves detection performance in adverse weather conditions by cascading fog or rain removal modules in front of the detector, or by directly enhancing the detector’s feature extraction capabilities [21,22]. Although these methods mitigate the impact of complex environments to some extent, most approaches still treat complex weather conditions as uniform visual noise in the spatial domain, and have not yet fully accounted for the fundamental differences in the physical degradation mechanisms between haze and rainfall.

**Figure 1:**
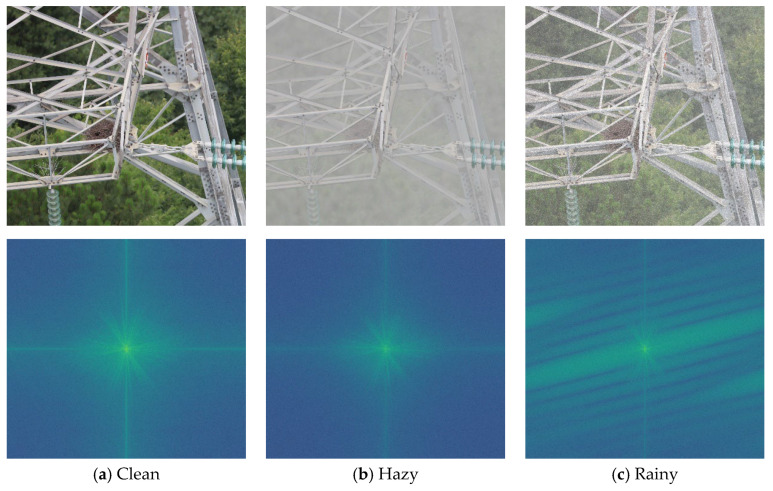
Comparison of spectral plots for clear-sky, hazy, and rainy images: (**a**) shows the spectral plot of the clear-sky image, with high-frequency details fully preserved; (**b**) shows the spectral plot of the hazy image, where high-frequency textures are suppressed; and (**c**) shows the spectral plot of the rainy image, exhibiting directional high-frequency noise. These physical characteristics inspired our frequency-domain decoupling strategy.

To investigate this physical essence, we have moved beyond the traditional spatial domain to conduct an in-depth analysis of the visual degradation caused by adverse weather conditions from the frequency domain. As shown in Figure 1, we compare the spectral plots of a clear power line image, a synthetic haze image, and a synthetic rain-affected image. It can be seen from Figure 1 that, compared to the clear image, haze causes attenuation of high-frequency signal energy at the periphery, with energy concentrated more in the central low-frequency region. This also illustrates that atmospheric scattering caused by haze is essentially a form of contrast attenuation, obscuring the texture of the target. In contrast, the spectral analysis of rain streaks not only fails to attenuate high-frequency components but actually amplifies high-frequency rays orthogonal to the physical direction of raindrops’ descent, constituting a form of destructive high-frequency noise.

There have already been some studies on the application of frequency-domain methods for object detection in adverse weather conditions. Chen et al. proposed a module called AStem, which formed a dual-path network by integrating a dual-attention mechanism with the Fast Fourier Transform (FFT), enabling the network to preserve more critical local details. Compared to traditional convolutional architectures, this approach improved detection accuracy by 2.7% in rainy and snowy conditions [23]. Chen et al. proposed the SMWG-DETR method, which is guided by Fourier spectral modulation and wavelets. This method first utilised the Fourier spectrum to extract key frequency components, then applied the Discrete Wavelet Transform (DWT) to low-level feature maps to obtain low- and high-frequency information, which in turn guided the upsampling process of high-level feature maps; this method demonstrated robust detection performance [24]. However, traditional transformation methods such as FFT and DWT lack translation invariance, making it difficult to preserve the structural phase information of the target without introducing artefacts. Furthermore, purely data-driven feature extractors suffer from a ‘black-box’ problem, lacking physical interpretability.

To address this issue, we propose the following hypothesis: if we can integrate physical prior knowledge of atmospheric within a translation-invariant complex wavelet domain, we can develop an efficient and interpretable solution for detecting foreign objects on power transmission lines in adverse weather conditions. Based on this concept, this paper proposes a novel, plug-and-play Physics-Prior Complex Wavelet Unrolling Decoupling Module (PCW-UDM). First, we utilise the two-dimensional double-tree complex wavelet transform (2D-DTCWT) to decouple degraded image features into low-frequency signals and complex high-frequency sub-band signals. The low-frequency signal branch suppresses global haze effects using a physics-guided algorithm for estimating transmittance and atmospheric light, whilst the high-frequency signal branch eliminates directional rain streaks by expanding the traditional iterative threshold contraction method into a learnable neural network.

The main contributions of this paper are summarised as follows:

(1) We propose a novel feature-level decoupling module, PCW-UDM, for the task of detecting foreign objects on power transmission lines under adverse weather conditions. This module seamlessly integrates signal processing theory with deep learning techniques, thereby effectively avoiding the semantic conflicts caused by traditional image restoration methods.
(2) We introduce the Physics-Guided Low-Frequency Dehazing (PGLD) branch and the LISTA-Unrolled High-Frequency Deraining (LUHD) branch. To the best of our knowledge, this marks the first instance of extending sparse optimisation algorithms to the complex wavelet domain, enabling robust detection under adverse weather conditions.
(3) We design a cross-domain consistency loss function. This ensures that the frequency amplitude and spatial features of images affected by adverse weather conditions remain consistent with those of their corresponding clear images, enabling the network to learn weather invariance.
(4) We have collected two synthetic datasets and conducted comprehensive comparative experiments and ablation studies on them. The experimental results demonstrate that our method outperforms current state-of-the-art detectors in the detection of foreign objects on power transmission lines under adverse weather conditions.

## 2. Related Work

### 2.1. Vision-Driven Power Line Inspection

In response to the growing demand for autonomous power grid inspection, a series of deep learning architectures have been developed to identify power line components and foreign objects. Early research primarily utilised a two-stage algorithm to first extract the target region and then perform classification and regression tasks. Huang et al. performed edge optimisation on Faster R-CNN, utilising the magnitude of image gradients to determine the amount of information contained therein, thereby filtering out effective features. This method effectively reduced the number of parameters in Faster R-CNN whilst maintaining the accuracy of foreign object detection [25]. Wang et al. adopted a cross-domain approach, employing a gradient-adaptive training strategy that utilised different gradients for the source and target domains. Building upon Faster R-CNN, they constructed a feature-adaptive alignment module capable of achieving local feature alignment across domains. This method achieved an 8.6% improvement in accuracy compared to baseline models for insulator defect detection [26]. Subsequently, single-stage algorithms, exemplified by YOLO, and Transformer models have found widespread application in the field of computer vision. Tang et al. proposed the ST2Rep-YOLOX method, which utilises the Swin Transformer V2 within the YOLOX backbone network to extract global features, whilst employing reparameterised VGG blocks to enhance feature extraction capabilities. This method demonstrated significant performance improvements over baseline models when detecting various types of foreign object intrusions [27]. Wu et al. proposed an emerging anchor-free detection method that effectively enhanced the network’s feature extraction capabilities for multi-scale objects by combining C2f with the SE attention mechanism, and introduced a lightweight detection head to improve the algorithm’s suitability for edge devices [28]. Liu et al. utilised improved masked image modelling for self-supervised training followed by fine-tuning on YOLO11, resulting in improved accuracy for the detection of minor defects in power transmission lines [29].

However, these advanced methods rely on aerial images being captured and trained under conditions of high visibility and clear weather. In adverse weather conditions, atmospheric scattering and occlusion by rain streaks can cause features to collapse further, making these purely data-driven models highly susceptible to disruption during deployment.

### 2.2. Object Detection Under Adverse Weather Conditions

To mitigate the impact of detection performance degradation caused by atmospheric degradation, the existing literature primarily falls into two paradigms: unsupervised domain adaptation (UDA) and cascaded recovery detection. The idea behind UDA methods is to use adversarial learning to ensure that the feature distributions between the source domain under clear weather and the target domain under adverse weather remain consistent across different scales and depths [17,30,31]. Li et al. proposed a novel domain-adaptive object detection framework, introducing an adversarial gradient inversion layer for complex object detection, which enabled in-depth adversarial analysis of challenging samples [32]. Notably, Guo et al. proposed a domain adaptation method that not only aligned the source and target domains at the image level but also accounted for semantic correlations between them, achieving state-of-the-art (SOTA) performance through alignment at both contextual and class-level semantic information [33]. Addressing the issue of spurious correlations generated by traditional image restoration techniques, Zhang et al. decoupled these correlations at both the instance and image levels, resulting in a method with improved robustness [34]. The cascade restoration–detection approach first enhances low-quality images using image enhancement techniques before feeding them into a detector for detection. Liu et al. proposed a detection algorithm named IA-YOLO, which first utilised a designed differentiable image processing module to adaptively enhance low-quality images before feeding them into a YOLOv3 detector for detection. This method could adaptively process images under both normal and adverse weather conditions, demonstrating excellent detection performance [35]. Wang et al. designed a dual-branch network that performed degradation modelling on degraded images to obtain multi-scale degraded representations. They then designed a multi-scale bidirectional feature fusion module for the restoration and detection branches, effectively resolving object detection issues under weather conditions such as rain, haze, and snow [36]. Liu et al. designed a remote sensing image detection method for hazy weather conditions, DFENet, comprising two branches and a dehazing module. To mitigate the impact of new noise introduced by the dehazing module, a haze prediction module was designed to dynamically adjust the feature weights of the two branches. The resulting model not only achieved excellent detection performance under hazy conditions but also improved detection performance under clear weather conditions [37].

Although the aforementioned methods have made commendable progress in object detection under various adverse weather conditions, the cascaded paradigm inherently suffers from severe semantic conflicts. Image restoration networks typically optimise loss using human visual perception metrics; however, this may lead to the loss of critical high-frequency edge features of certain objects, and artefacts generated during the restoration phase are prone to being amplified by subsequent detection heads. Similarly, UDA methods often treat weather degradation as ‘black-box’ noise, without fundamentally analysing the attenuation mechanisms of rain and haze from a physical perspective, thereby limiting their generalisation capabilities in complex environments.

### 2.3. Application of Frequency-Domain Methods in Object Detection Tasks

Recent research indicates that mapping spatial images to the frequency domain can provide a robust alternative for separating background information from adverse weather noise [38,39,40]. Unlike spatial convolution, the frequency domain essentially decouples global illumination variations from burst noise. Lyu et al. proposed a pure Fourier transform model that employed a bimodal Fourier representation for RGB-T data, and designed a frequency-decomposed edge-aware module capable of performing deep decomposition and filtering of the Fourier components of low-level features; this method effectively improved the performance of salient object detection [41]. Gakhar et al. proposed a Fourier-domain adaptive method that replaces the low-frequency components of the source-domain image with those of the target-domain image, enabling the model to better cope with domain shift phenomena caused by adverse weather conditions [38]. Liu et al. proposed a multimodal image fusion method that utilised a designed wavelet-space state module to perform decoupled multi-frequency degradation in the wavelet domain, modelling high-frequency degradation across different directions to enhance the model’s generalisation capability under complex weather conditions [42]. Gao et al. proposed a Transformer-based image restoration method. By introducing wavelet-based prior information to construct a frequency-guided Transformer encoder to guide the extraction of image features, they effectively improved the restoration quality of low-quality images [43].

However, when addressing aerial object detection tasks with high accuracy requirements, traditional frequency-domain transformation methods face inherent limitations due to their physical characteristics. The FFT inherently lacks spatial locality, making it difficult to spatially align regional targets within the reconstructed feature domain [44,45]. Whilst the DWT possesses spatial-frequency locality, it suffers from a severe lack of translation invariance and has limited directional selectivity. Research in fundamental signal processing indicates that the critical downsampling in DWT can produce severe aliasing and checkerboard artefacts even with minor spatial shifts; the resulting noise is highly detrimental to subsequent detection stages in image processing [46,47,48].

To overcome these inherent structural limitations, the dual-tree complex wavelet transform (DTCWT) has emerged in the field of fundamental signal processing research [49,50]. By employing two parallel DWT trees, the DTCWT ingeniously achieves near-perfect translation invariance and provides enhanced multi-directional selectivity. Building upon these mathematical foundations, recent research has demonstrated that combining DTCWT with deep convolutional neural networks enables the effective extraction of multi-frequency generalised features, thereby inherently suppressing complex environmental interference without sacrificing critical local details [51,52,53].

## 3. Methodology

To address the challenge of robust detection of foreign objects on power transmission lines in rainy and hazy conditions, this paper proposes a Physics-Prior Complex Wavelet Unrolling Decoupling Module (PCW-UDM). Rather than performing pixel-level restoration on traditional images, this module is embedded as a plug-and-play feature component within multi-scale features. As shown in Figure 1, the PCW-UDM ingeniously utilises the translational invariance and multi-directional selectivity of the B-tree complex wavelet transform to decouple the complex visual degradation problem into low-frequency haze signals and high-frequency rain streak signals, which are then reconstructed using corresponding physical prior models and deep expansion algorithms.

### 3.1. Overall Network Architecture

**Figure 2:**
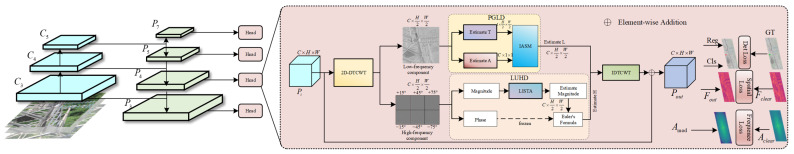
Overall network architecture. The backbone consists of a ResNet-50 network and an FPN module for multi-scale feature extraction. Low-frequency and high-frequency signals are obtained via 2D-DTCWT and fed into the PGLD and LUHD, respectively, and the extracted corresponding clean signals are then connected via inverse transforms and a residual structure.

As shown in Figure 2, the left-hand side illustrates the multi-scale feature extraction scheme, which employs the classic ResNet50 as the backbone module and a feature pyramid to extract multi-scale semantic feature maps. For images containing rain and haze, these feature maps contain strong atmospheric attenuation and directional rain streak noise; therefore, we embed the proposed PCW-UDM into the feature processing stage of the deep network.

Specifically, the feature maps generated at each scale are fed into the PCW-UDM. The PCW-UDM first employs a 2D DWDCT to downsample the feature maps whilst decomposing them into a low-frequency component and six complex high-frequency components in different directions. The generated low-frequency signal is fed into the PGLD module, which first estimates the spatial transmittance map and the global atmospheric light vector in parallel before inputting these into the inverse atmospheric scattering model to derive an estimated clear low-frequency background; the generated high-frequency signal is then fed into the LUHD module, where each angle is decomposed into amplitude and phase signals. Here, the phase signal is frozen and bypassed, whilst the amplitude signal is fed into an iterative thresholding algorithm with deep unfolding to obtain a clean high-frequency amplitude signal stripped of rain streak noise. This is then combined with the phase signal using Euler’s formula to generate new high-frequency edges. Finally, the low-frequency and high-frequency signals obtained from these two modules are transformed back into the spatial domain via a dual-tree complex wavelet inverse transform. They are then superimposed onto the original input features via residual interpolation to produce the final feature map, which is fed into the detection head.

### 3.2. Physics-Guided Low-Frequency Dehazing (PGLD)

The 2D-DTCWT procedure involves downsampling and filtering real and imaginary trees based on the input’s features at different scales, yielding four sets of real sub-bands $\{LL,LH,HL,HH{\}}^{a}$ and $\{LL,LH,HL,HH{\}}^{b}$. The low-frequency components ensure translation invariance by taking the average of the two trees:

(1) $$L=\frac{1}{2}\left(LL^{a}+LL^{b}\right)$$

The high-frequency components are combined directionally to form complex coefficients:

(2) $$\begin{array}{c} H_{1}=LH^{a}+i\cdot LH^{b}(+15^{\circ }),\\ H_{2}=HL^{a}+i\cdot HL^{b}(+45^{\circ }),\\ H_{3}=HH^{a}+i\cdot HH^{b}(+75^{\circ }),\\ H_{4}=LH^{b}+i\cdot LH^{a}(-15^{\circ }),\\ H_{5}=HL^{b}+i\cdot HL^{a}(-45^{\circ }),\\ H_{6}=HH^{b}+i\cdot HH^{a}(-75^{\circ }), \end{array}$$

where *i* is the imaginary unit.

The low-frequency signal $L\in R^{C\times \frac{H}{2}\times \frac{W}{2}}$ obtained after 2D-DTCWT decoupling retains the image’s global illumination and contrast attenuation caused by haze. As shown in Figure 1, haze primarily causes a shift in concentration of low-frequency energy whilst suppressing high-frequency signals. Therefore, we have not employed conventional black-box convolution for fitting here, instead utilising a classical atmospheric scattering model introduced into the feature space to physically model the degradation process:

(3) $$L=L_{clean}⊙T+A⊙\left(1-T\right)$$

where $L_{clean}$ denotes the clean low-frequency features we aim to obtain, *A* represents the global atmospheric light feature, T is the spatial transmittance map, and ⊙ denotes element-wise multiplication.

To solve this inverse problem, PGLD employs two parallel lightweight parameter estimation sub-networks to estimate T and A, and then reconstructs the clean features via the inverse atmospheric scattering model.

(1) Estimate T

**Figure 3:**
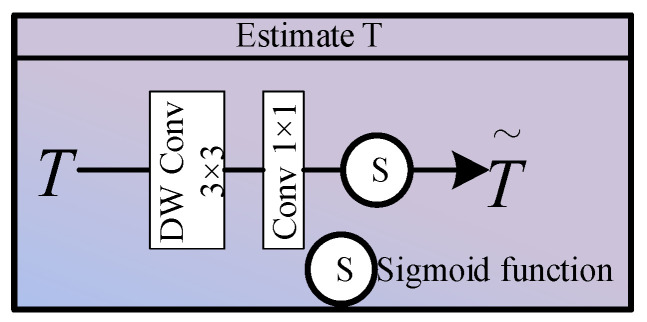
Block diagram of the Estimate T module.

As shown in Figure 3, in the Estimate T module, we first feed the low-frequency features into this sub-network. To maintain a local receptive field whilst significantly reducing computational complexity, we first apply a 3 × 3 deep separable convolution, followed by 1 × 1 pixel-wise convolution to perform cross-channel dimensionality reduction, and finally we normalise the output range using a Sigmoid activation function:

(4) $$T=\sigma \left({Conv}_{1\times 1}({DWConv}_{3\times 3}(L))\right)\in R^{1\times \frac{H}{2}\times \frac{W}{2}}$$

where DWConv denotes deep separable convolution and σ(·) denotes the Sigmoid activation function.

(2) Estimate A

**Figure 4:**
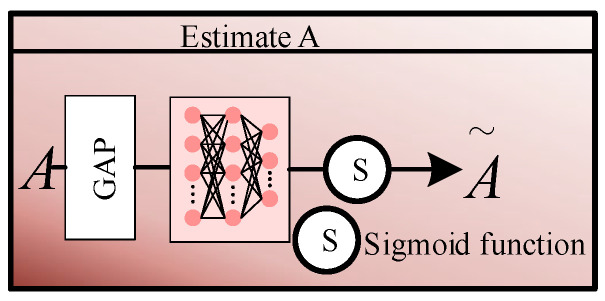
Block diagram of the Estimate A module.

As shown in Figure 4, in the Estimate A module, we first use global average pooling to compress the spatial dimensions from $\frac{H}{2}\times \frac{W}{2}$ to 1×1, thereby extracting global information. This vector is then fed into a two-layer multi-layer perceptron to facilitate high-order non-linear interactions between channels, followed by normalisation using the Sigmoid activation function:

(5) $$A=\sigma \left(MLP(GAP(L))\right)\in R^{C\times 1\times 1}$$

where GAP(·) denotes global average pooling.

(3) Inverse Atmospheric Scattering Model Reconstruction (IASM)

After obtaining the clean transmittance map *T* and atmospheric light vector *A* through the above steps, the IASM module is employed to generate the purified low-frequency features $\tilde{L}$.

(6) $$\tilde{L}=\frac{L-A⊙(1-T)}{max(T,t_{0})}\in R^{C\times \frac{H}{2}\times \frac{W}{2}}$$

Here, $t_{0}$ represents a very small lower bound for the constant, primarily to prevent T from tending towards 0 when haze concentration is extremely high, which would cause a division-by-zero error in the above equation.

Through the proposed PGLD branch, the network can eliminate low-frequency haze interference under strong supervision without requiring any real reference clear images, relying solely on the hard constraints imposed by physical equations.

### 3.3. LISTA-Unrolled High-Frequency Deraining (LUHD)

The six high-frequency components $H_{d}\in C^{C\times \frac{H}{2}\times \frac{W}{2}}(d\in \{\pm 15^{\circ },\pm 45^{\circ },\pm 75^{\circ }\})$ obtained after 2D-DTCWT decomposition not only contain edge information of foreign objects but also incorporate directional high-frequency noise generated by heavy rain. Conventional spatial-domain convolution struggles to filter out these rain streaks without blurring the edges; therefore, we have designed the LUHD branch to remove rain streaks whilst preserving edges in the complex polar domain. This involves the following steps.

(1) Magnitude–Phase Separation and Phase Freezing

The essence of complex high-frequency features is that the magnitude represents the intensity of the edge, whilst the phase records the spatial position and orientation of the edge. As shown in the LUHD module in Figure 2, we first convert the complex tensor $H_{d}$ for each direction into polar coordinates by extracting its magnitude and phase:

(7) $$M_{d}=\sqrt{(R(H_{d}){)}^{2}+(I(H_{d}){)}^{2}}\in R^{C\times \frac{H}{2}\times \frac{W}{2}}$$

(8) $$P_{d}=arctan\left(\frac{I(H_{d})}{R(H_{d})}\right)\in R^{C\times \frac{H}{2}\times \frac{W}{2}}$$

To avoid introducing any spatial shifts or geometric distortions during the rain removal process, we freeze the phase information so that it does not participate in training. As indicated by the dotted arrows in Figure 2, the phase is bypassed and does not contribute to the update of any network parameters; all rain removal operations are applied solely to the contaminated magnitude.

(2) LISTA-Unrolled Magnitude Purification

As can be seen from Figure 1, rain streaks exhibit high sparsity in specific high-frequency information. Inspired by this, we model the process of separating clean edges from the contaminated magnitude as a classical sparse coding LASSO optimisation problem:

(9) $$\underset{E_{d}}{min}\;\frac{1}{2}∥M_{d}-E_{d}{∥}_{2}^{2}+\lambda ∥\Psi \left(E_{d}\right){∥}_{1}$$

**Figure 5:**
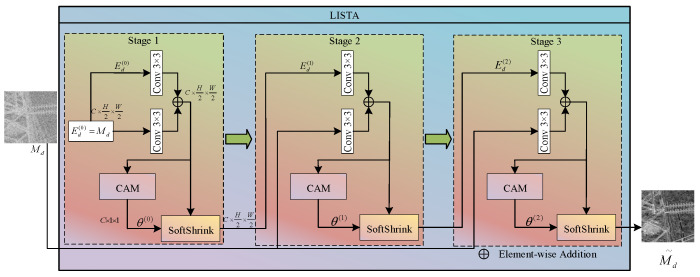
Block diagram of the LISTA module. This process applies only to the amplitude information of the high-frequency components; the phase information is frozen and does not participate in the training process.

Since traditional iterative shrinkage algorithms require a significant amount of time to terminate the iteration when solving this optimisation problem, they cannot be applied to the engineering context of UAV inspections. We therefore introduce deep unrolling techniques here, unfolding the mathematical iteration process into a learnable feedforward neural network comprising K layers. The specific structure is shown in Figure 5.

**Figure 6:**
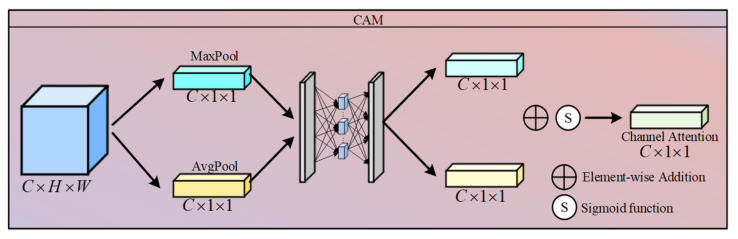
Block diagram of the CAM network.

Setting the initial state as $E_{d}^{\left(0\right)}=M_{d}$, the update of the amplitude tensor in the *k*-th expansion layer of the LISTA module strictly follows the following formula:

(10) $$Z_{d}^{\left(k\right)}={Conv}_{A}^{\left(k\right)}\left(E_{d}^{\left(k-1\right)}\right)+{Conv}_{B}^{\left(k\right)}\left(M_{d}\right)$$

(11) $$E_{d}^{\left(k\right)}=SoftShrink\left(Z_{d}^{\left(k\right)},\theta^{\left(k\right)}\right)$$

where ${Conv}_{A}^{\left(k\right)}$ and ${Conv}_{B}^{\left(k\right)}$ denote the learnable 3 × 3 convolutional filters in the *k*-th layer, which replace the predefined analytical dictionary matrix required in traditional algorithms; and SoftShrink(x,θ)=sgn(x)⋅max(|x|−θ,0) is a non-linear soft-threshold activation function, in which $\theta^{\left(k\right)}$ is an adaptive threshold parameter learned via a Connection Attention Module (CAM) through a $Z_{d}^{\left(k\right)}$ channel, allowing for dynamic adaptation to the intensity of rain streaks in different image regions. The specific CAM structure is shown in Figure 6.

After K layers of expansion, the network outputs a clean amplitude estimate ${\tilde{M}}_{d}$ that has been stripped of rain streak noise. ${\tilde{M}}_{d}$ is then combined with the previously frozen original phase $P_{d}$ using Euler’s formula to reconstruct the clean high-frequency information component in the complex plane, as shown in the following formula:

(12) $${\tilde{H}}_{d}={\tilde{M}}_{d}⊙e^{jP_{d}}={\tilde{M}}_{d}⊙(cos(P_{d})+j\cdot sin(P_{d}))$$

Finally, the $\tilde{L}$ output from the PGLD branch and the six directional ${\tilde{H}}_{d}$ components output from the LUHD branch are jointly fed into a two-dimensional dual-tree complex wavelet inverse transform to restore the resolution of the original space, and the final features are obtained via residual interpolation:

(13) $$P_{rec}=IDTCWT\left(\tilde{L},{\tilde{H}}_{1},{\tilde{H}}_{2},\ldots ,{\tilde{H}}_{6}\right)\in R^{C\times H\times W}$$

(14) $$P_{out}={Conv}_{1\times 1}(F_{rec})+P_{in}\in R^{C\times H\times W}$$

### 3.4. Joint Loss Function

To ensure that the PCW-UDM proposed in this paper can effectively decouple contaminated images and that the reconstructed features can serve the downstream detection head, we have designed a spatio-temporal cross-domain consistency optimisation strategy comprising three components: the object detection loss $L_{det}$, the spatial feature consistency loss $L_{spatial}$, and the frequency-domain amplitude consistency loss $L_{freq}$.

(15) $$L_{total}=L_{det}+\lambda_{1}L_{spatial}+\lambda_{2}L_{freq}$$

where λ_1_ and λ_2_ are the weight hyperparameters for $L_{spatial}$ and $L_{freq}$, respectively. In this paper, the parameters are set to 0.2 and 0.05.

(1) Spatial Consistency Loss

As real-world adverse weather images lack paired clean images to serve as supervision, we utilise a frozen teacher network during the training phase to extract features $F_{clear}$ from clear images, which can serve as the ideal constraint benchmark for the spatial dimension. For the purified features $F_{out}$ reconstructed by the PCW-UDM from the teacher network, we calculate the Euclidean distance between the two using the mean squared error. This loss function ensures that the image features after haze or rain removal align spatially and semantically with those of the clean image:

(16) $$L_{spatial}=\frac{1}{CHW}||F_{out}-F_{clear}|{|}_{2}^{2}$$

(2) Frequency-domain consistency loss

Relying solely on spatial absolute value constraints makes it difficult to detect residual low-frequency background signals from incomplete dehazing and residual high-frequency micro-oscillations from rain removal. We therefore innovatively introduce a frequency-domain alignment mechanism, utilising a two-dimensional Fast Fourier Transform (FFT) to map spatial features to frequency-domain features and obtain the corresponding amplitude spectrum:

(17) $$A_{mod}=log(1+|F(F_{out})|)$$

(18) $$A_{clear}=log(1+|F(F_{clear})|)$$

To ensure the network can accurately remove the directional high-frequency signals caused by rain streaks in the frequency domain, we constrain the modulated $A_{mod}$ to converge towards $A_{clear}$. Here, we have chosen the $L_{1}$ norm over the $L_{2}$ norm, as the $L_{1}$ norm exhibits greater robustness to high-frequency fluctuations [54].

(19) $$L_{freq}=\frac{1}{CHW}∥A_{mod}-A_{clear}{∥}_{1}$$

(3) Object Detection Loss

The object detection loss comprises bounding box regression loss and classification loss:

(20) $$L_{det}=L_{cls}+L_{reg}$$

where $L_{cls}$ and $L_{reg}$ represent the classification loss and regression loss, respectively.

## 4. Experiments and Analysis

### 4.1. Datasets and Implementation Details

#### 4.1.1. Datasets

In real-world power line scenarios, it is difficult to obtain paired aerial images showing both adverse weather conditions and clear conditions from the same viewpoint; consequently, this work is currently carried out using physical models for synthesis. Following this approach, this paper has generated two datasets: the Single-Adverse Dataset and the Multi-Adverse Dataset.

Single-Adverse Dataset: This dataset was primarily sourced from publicly available material on the internet and images captured by our own drones. A total of 1000 images were compiled, with a training:test:validation ratio of 8:1:1. This dataset contains only one type of foreign object: bird nests.

Multi-Adverse Dataset: This dataset was also sourced from the internet and captured using drones, resulting in 4500 images. The dataset is divided into training, test, and validation sets in a ratio of 8:1:1, and contains four types of foreign objects: bird nests, kites, balloons, and rubbish.

**Figure 7:**
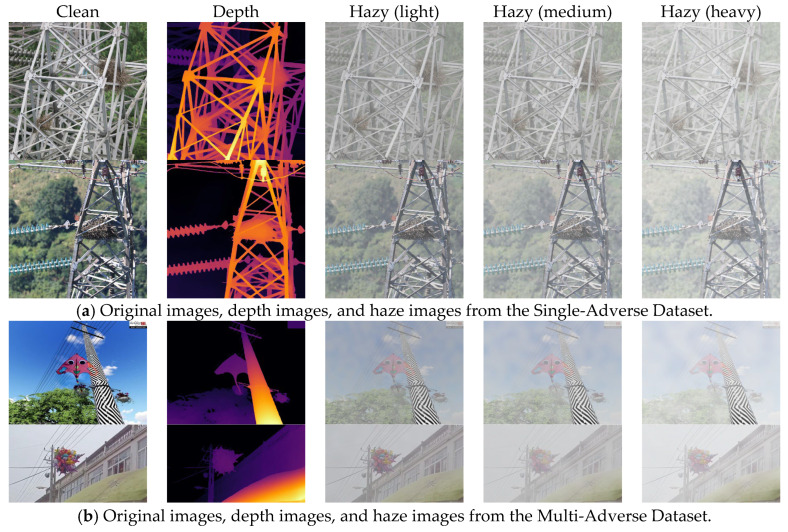
Composite images of haze showing different concentrations of haze across the two datasets, where (**a**) represents the Single-Adverse Dataset and (**b**) represents the Multi-Adverse Dataset.

For the synthesis of haze images, we selected a classic atmospheric scattering model, as shown in Equation (1), where $T=e^{-\beta d}$ and β is the atmospheric scattering coefficient. Following the work of Feng et al., we selected $\tilde{A}∼\tilde{N}(0.8,0.05^{2})$ and $\beta ∼N(0.045,0.02^{2})$ [55]. We employed the Depth Anything v2 algorithm proposed by Yang et al. for monocular depth estimation [56]. Figure 7 displays the synthetic haze images generated for the two datasets at different haze concentrations.

**Figure 8:**
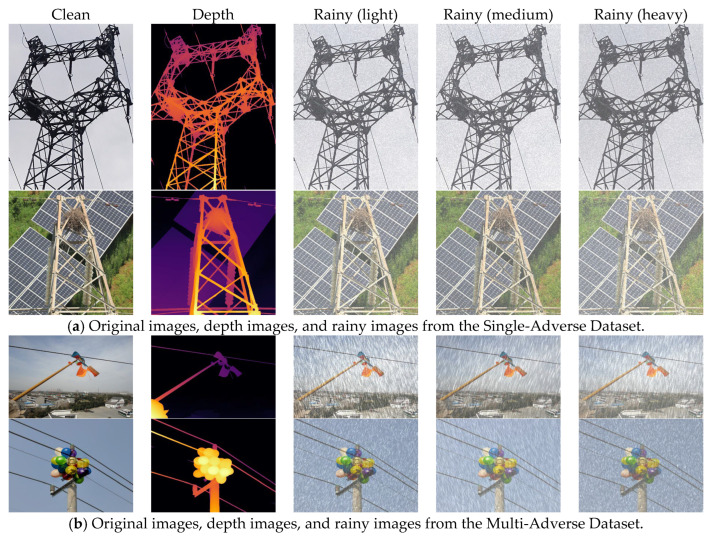
Composite images of rain showing different concentrations of rain across the two datasets, where (**a**) represents the Single-Adverse Dataset and (**b**) represents the Multi-Adverse Dataset.

For the synthetic images of rainy weather, real-world rainfall is often accompanied by water vapour. We first generate a water vapour background accompanying the rainfall using the depth map and a scattering coefficient within a specified range. We then generate random noise using a predefined raindrop density distribution and apply motion blur kernels of varying sizes and descent angles, thereby generating highly directional high-frequency rain streaks. Figure 8 shows examples of the synthetic rainy-day images generated for the two datasets.

#### 4.1.2. Implementation Details

The method proposed in this paper and all comparison methods were evaluated under a unified experimental setup, with FCOS selected as the baseline detector and ResNet50 as the backbone network; the input image size was set to 640 × 640. We followed the standard ‘schedule_1x’ training configuration from MMDetection, with all models trained for 12 epochs. The training process utilised SGD, with an initial learning rate set to 0.01. The experiments in this paper were implemented on a workstation equipped with a single NVIDIA RTX 4090 (24GB VRAM) GPU, using the PyTorch 2.0 framework.

Regarding the key module parameters of the proposed method, the LISTA expansion step count was set to three, and the weights for the spatial consistency loss and frequency-domain consistency loss were set to 0.2 and 0.05, respectively. The teacher network acquires features at various scales from the clean dataset, whilst the remaining components do not participate in any training.

To verify the robustness of this algorithm against the inherent variance in deep learning, we report quantitative results obtained from three independent runs of key comparison methods, each using a different random seed. These results are presented in the form of ‘mean ± standard deviation’. Details are shown in Table 1 and Table 2.

### 4.2. Comparison with State-of-the-Art

We selected FCOS as the baseline detection framework. Based on current state-of-the-art strategies, we categorised existing methods into the following three classes: (1) Direct—no image restoration or enhancement is performed, and the detector is used directly for training and testing. This strategy includes Baseline-1, trained solely on clear weather data, and Baseline-2, trained on mixed weather data. (2) Separate—a separated cascade paradigm of restoration followed by detection is adopted. This paper selects the state-of-the-art dehazing network FFA-Net [57] and the deraining network MPRNet [58] as enhancement modules, respectively, and then cascades them with FCOS to form two sets of comparison methods. (3) Union—adverse weather feature enhancement is jointly optimised with the object detection task. This category includes IA-YOLO and DS-Net, which are designed for adverse weather, as well as the proposed PCW-UDM+FCOS. All experiments employ mAP@0.5 as the evaluation metric, reporting the detection accuracy of various methods on the clean, hazy, and rainy test sets.

#### 4.2.1. Results on Single-Adverse Dataset

Table 1 presents a comparison of our proposed method with several other state-of-the-art methods. It is worth noting that, as the Single-Adverse Dataset contains only one type of foreign object (bird nests), the overall scenes are relatively simple, and the dataset consists predominantly of images featuring bird nests; consequently, the overall performance of different methods on this dataset is relatively high.

As can be seen from Table 1, adverse weather conditions have a significant impact on object detection. Baseline-1, trained solely on the sunny dataset, achieved a precision of 91.5% on the clean dataset; however, due to atmospheric degradation, its performance on the hazy and rainy test sets dropped to 72.8% and 75.1%, respectively. This indicates that without supervision from adverse weather samples, the features learned by this model are unable to accurately detect objects in images with reduced contrast and directional stripe interference. Baseline-2, trained on mixed weather data, improved to 80.9% and 82.4% under hazy and rainy conditions, respectively, suggesting that incorporating adverse weather samples into training can mitigate the domain shift issue to some extent. However, the detection accuracy of this approach under clean conditions dropped to 88.6%, which is lower than Baseline-1′s 91.5%, indicating that simply using data mixing for robustness training comes at the expense of some detection performance under normal weather conditions.

The Separate strategy did not perform well here, as the Sep-1 model achieved an accuracy of 81.6% under hazy conditions, which is only 0.7% higher than Baseline-2. The Sep-2 model achieved 81.2% under rainy conditions, which is not as effective as the Union method. This suggests that the separate paradigm of restoration followed by detection can improve image visibility to some extent, but the image restoration process may over-smooth image edges and texture structures, leading to a decline in detection performance under clean conditions.

The Union strategy demonstrated superior balance, as IA-YOLO and DS-Net outperformed the Separate methods under all three weather conditions. Although there was still some decline in accuracy under clean conditions, accuracy was significantly improved under hazy and rainy conditions, indicating that jointly optimising feature enhancement and the detection process can effectively mitigate the semantic drift caused by rainy and hazy weather. The PCW-UDM+FCOS method proposed in this paper achieved the best results across all conditions, reaching 91.2%, 88.1%, and 87.4% on the clean, hazy, and rainy test sets, respectively. Compared with the state-of-the-art runner-up method, DS-Net, our method achieved improvements of 3.3 and 2.1 percentage points under hazy and rainy conditions, respectively. The minimal standard deviation further proves that these gains are inherently driven by our architectural design rather than training variance. It should be noted that although the proposed method exhibits some performance degradation under adverse weather conditions, the extent of this decline is significantly smaller than that of other methods, indicating that the proposed method is more effective at extracting stable structural information that is unaffected by weather interference.

#### 4.2.2. Results on Multi-Adverse Dataset

The Multi-Adverse Dataset contains four types of foreign objects, resulting in greater background complexity and larger scale contrast. Consequently, on this dataset, adverse weather conditions not only degrade the image features of the target itself but also introduce background interference, making detection more challenging. Table 2 presents the experimental results of each method on this dataset.

As can be seen from Table 2, when the detection task is expanded from single-class to multi-class, the detection performance of all models declines significantly, indicating that the detrimental impact of adverse weather on detectors is amplified in complex scenarios. The Baseline-1 model maintained a high detection accuracy of 88.7% under clean conditions, but achieved only 61.4% and 64.2% under hazy and rainy conditions, respectively, demonstrating a marked decline in performance. This indicates that complex multi-class scenarios severely impair the generalisation of models trained under clear conditions to adverse weather conditions. Baseline-2, through mixed-weather training, achieved a partial improvement in accuracy under hazy and rainy conditions, rising to 73.8% and 75.1%, respectively; however, its performance under clean conditions still dropped to 84.6%, indicating that the data mixing strategy struggles to strike a balance between different weather domains.

The Separate strategy exhibits greater limitations in multi-class scenarios, with Sep-1 and Sep-2 achieving average accuracies of only 74.0% and 74.3%, respectively, which is lower than Baseline-2. This suggests that in multi-class object detection tasks, image restoration tends to treat key textures of certain classes as noise and remove them, resulting in a decline in the detector’s ability to distinguish between different objects.

The performance of IA-YOLO and DS-Net under the Union strategy was generally superior to that of Direct and Separate. Specifically, DS-Net achieved 83.4%, 79.6%, and 80.2% under the three weather conditions, indicating that end-to-end joint optimisation can more effectively coordinate the relationship between adverse weather modelling and object detection. The method proposed in this paper also performs best in multi-class adverse weather scenarios, achieving 86.5%, 83.8%, and 84.1% under clean, hazy, and rainy conditions, respectively. The lead is particularly pronounced in rainy and hazy conditions, indicating that the proposed method can effectively separate low-frequency haze degradation from high-frequency rain streak interference in complex scenarios, significantly enhancing the stable recognition capability of multi-class objects.

#### 4.2.3. Qualitative Visual Analysis

**Figure 9:**
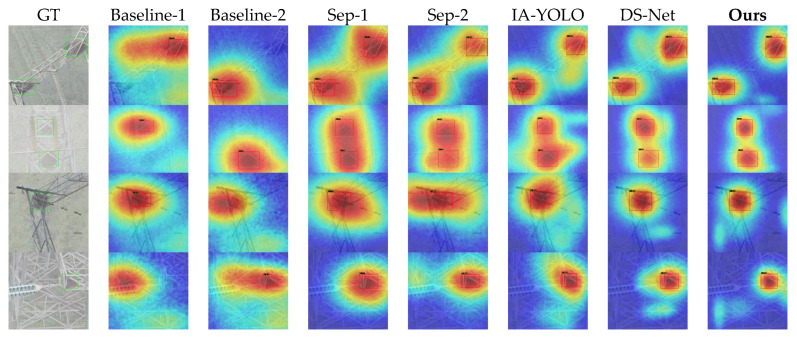
Visual comparison of various methods on the Single-Adverse Dataset. The second and third rows show detection under hazy conditions, whilst the fourth and fifth rows show detection under rainy conditions.

**Figure 10:**
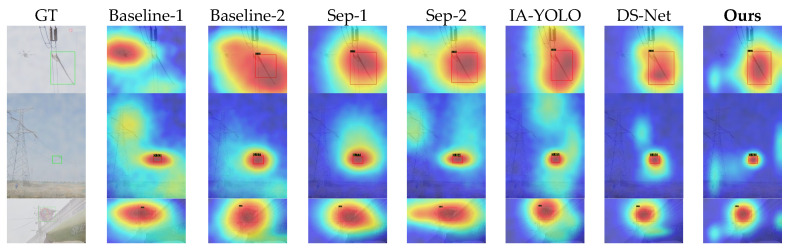

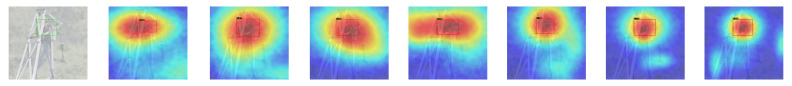
Visual comparison of various methods on the Multi-Adverse Dataset. The second and third rows show detection under hazy conditions, whilst the fourth and fifth rows show detection under rainy conditions.

Figure 9 and Figure 10 demonstrate the qualitative visual results of various methods across the two datasets. The first column of each figure shows the corresponding hazy or rainy image and its ground-truth bounding box, whilst the remaining columns display the corresponding response heatmaps for each method. The detection results also present the predicted bounding boxes and confidence scores.

As can be seen from Figure 9, some samples in Baseline-1 and Baseline-2 exhibit obvious false positives or false negatives, with significant shifts in their heatmaps and generally low confidence scores. Sep-1, Sep-2, IA-YOLO, and DS-Net are able to enhance the response in the target region to some extent, but the overall response remains somewhat dispersed. Furthermore, as the high-response regions in some samples extend into the surrounding background, the improvement in detection confidence is limited. The method proposed in this paper demonstrates a more concentrated and distinct response near the target region, with relatively high confidence scores.

As can be seen from Figure 10, as the number of classes and background complexity increase, the differences between the various methods become more pronounced. Overall, all methods are affected to varying degrees.

The Baseline method is most severely affected, with very weak target responses and significant localisation shifts. The Separate method still manages to improve performance effectively; however, when other objects appear in the background—such as drones—the model’s responses are easily disrupted, leading to heatmap diffusion and low confidence. IA-YOLO and DS-Net perform slightly better, but still suffer from insufficient feature concentration and low confidence scores. In contrast, our model achieves the best results in terms of both region localisation and confidence scores, demonstrating that our method can strongly suppress complex backgrounds and effectively mitigate the interference caused by rainy and hazy weather and complex backgrounds on feature representation.

**Figure 11:**
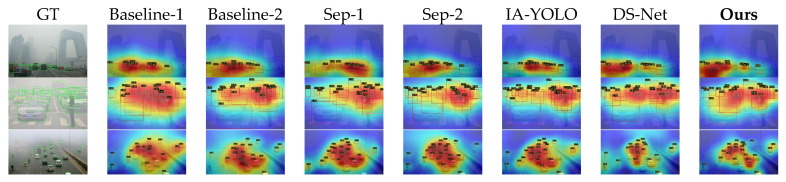
Visual comparison of various methods on the real-world RTTS dataset.

Furthermore, to evaluate the performance of our algorithm in real-world adverse weather scenarios, we conducted qualitative experiments using a subset of the Real-Time Traffic Scenarios (RTTS) dataset [60]. The specific test results are shown in Figure 11. As can be seen from Figure 11, when faced with real-world environments, the Direct strategy is significantly affected by image degradation caused by atmospheric scattering; although the overall features do not undergo major shifts, false positives and false negatives occur in most cases. The Separate strategy mitigates this situation to some extent, but it tends to concentrate features on high-confidence targets rather than distributing weights across each target. IA-YOLO and DS-Net also demonstrate strong robustness in real-world scenarios, successfully detecting the majority of targets; however, they still exhibit excessive shifts in heatmap features. Our method demonstrates the best robustness, as the introduction of the PCW-UDM module enables the model to restore the original frequency-domain features of the image to the greatest extent possible when faced with image degradation, resulting in the most balanced heatmap features and generally high confidence levels.

### 4.3. Robustness Analysis Under Different Weather Conditions

**Figure 12:**
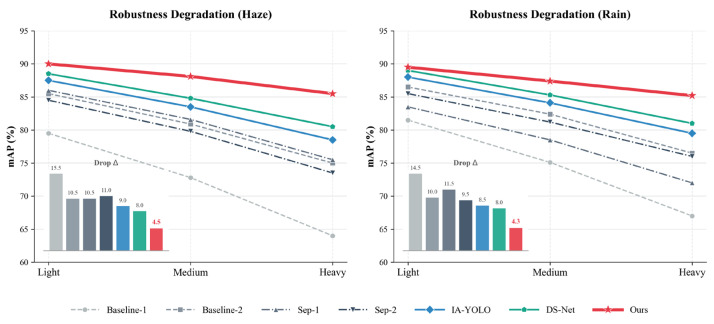
Comparison of robustness results for different methods on the Single-Adverse Dataset. The left panel shows detection performance under different levels of haze; the right panel shows detection performance under different levels of rainfall.

Figure 12 illustrates a comparison of detection performance on the Single-Adverse Dataset under varying degrees of image degradation. Three scenarios—mild, moderate, and severe—were constructed by altering the atmospheric light intensity and scattering coefficient for haze and rainfall, respectively.

As shown in Figure 12, the Baseline-1 model in the Direct category exhibits a significant decline in performance under both types of weather degradation. Under hazy conditions, detection accuracy dropped from 79.5% in the mild scenario to 72.8% in the moderate scenario, and suffered a catastrophic decline to 64.0% in the severe scenario; under rainfall conditions, detection accuracy also exhibited a sharp decline. This indicates that models trained on clear weather data are highly sensitive to adverse weather conditions, and their detection capabilities deteriorate rapidly when images exhibit more severe haze or directional rain streaks. In contrast, the Baseline-2 model mitigated detection performance to some extent; however, under severe hazy and rainy conditions, accuracy still only reached 75.0% and 76.5%, respectively. This suggests that whilst it can alleviate some domain shift issues, it remains unsuitable as a general-purpose detection model.

The Sep-1 and Sep-2 methods within the Separate strategy still demonstrate relatively good detection performance under mild weather degradation; however, as weather conditions worsen, their performance declines rapidly. This indicates that the separated image enhancement methods can improve image visibility to a certain extent, but this improvement remains largely confined to the visual level, making it difficult to maintain stable detection capabilities under severely degraded conditions.

The methods within the Union strategy generally demonstrate greater stability. IA-YOLO and DS-Net exhibit significantly less performance degradation under heavy hazy and rainy conditions compared to the Direct and Separate strategies. Specifically, DS-Net’s accuracy drops from 88.5% to 80.5% under hazy conditions and from 89.0% to 81.0% under rainy conditions, demonstrating overall robust performance. This demonstrates that end-to-end joint optimisation of feature enhancement tasks can effectively mitigate performance variations caused by severe weather conditions.

The method proposed in this paper achieves optimal results under both hazy and rainy conditions. In hazy scenarios, the method achieves accuracy rates of 90.0%, 88.1% and 85.5%; conversely, in rainy conditions, our method achieved 89.5%, 87.4% and 85.2%, outperforming other state-of-the-art methods in terms of both accuracy and the magnitude of performance degradation. This demonstrates that as weather degradation intensifies, the advantage of our method over other approaches gradually increases.

### 4.4. Ablation Studies

In this section, we conduct ablation studies on our model using the Single-Adverse Dataset, focusing on different modules. This primarily includes: (1) combinations of different modules within PCW-UDM; (2) a comparison of the effectiveness of consistency loss and different weights; and (3) an ablation comparison of different frequency-domain methods.

(1) Analysis of the effectiveness of different modules in PCW-UDM

Table 3 illustrates the contribution of different modules in PCW-UDM to the model’s detection performance under adverse weather conditions. Detection performance is relatively low when using the baseline model. After introducing PGLD, the model’s detection accuracy in haze scenarios improves significantly, indicating that the low-frequency degradation modelling branch can effectively mitigate the problem of global contrast reduction caused by haze. The introduction of LUHD improves the model’s performance in rainy conditions, indicating that the high-frequency decoupling branch effectively suppresses directional noise interference introduced by rain streaks. When both PGLD and LUHD are introduced simultaneously, the model’s performance in hazy and rainy conditions shows a slight decline; however, its overall average performance still improves significantly, and the two modules exhibit a certain degree of complementarity in terms of weather degradation.

(2) Effectiveness of the Consistency Loss and Comparison of Different Weights

Table 4 illustrates the contribution of the consistency loss function to detection accuracy. As can be seen from the table, when using only the detection loss, the model achieved detection accuracies of 90.3%, 86.7%, and 86.3% under clean, hazy, and rainy conditions, respectively. After introducing $L_{spatial}$, the improvement under hazy conditions is particularly pronounced, with an increase of 0.9%, indicating that this loss effectively promotes semantic alignment between degraded and clear features. Upon introducing $L_{freq}$, the accuracy under rainy conditions improves by 0.7%, indicating that this loss function can suppress frequency interference caused by rain streaks. The model achieves optimal performance when both consistency loss functions are applied together, demonstrating that spatial-domain constraints and frequency-domain constraints complement each other well.

**Figure 13:**
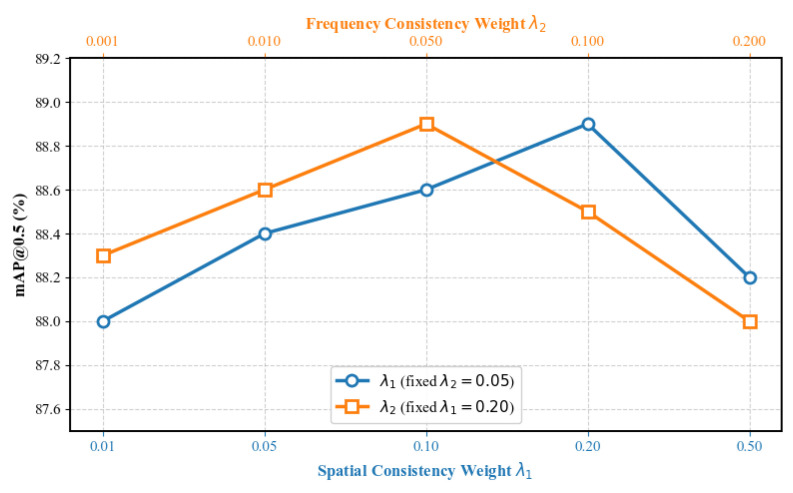
Hyperparameter sensitivity analysis plot for $L_{spatial}$ and $L_{freq}$.

Additionally, a hyperparameter sensitivity analysis was conducted for the weights of the two losses, with the specific results shown in Figure 13. The analysis employed the method of controlled variables by varying $\lambda_{1}$ whilst keeping $\lambda_{2}$ fixed at 0.05, and varying $\lambda_{2}$ whilst keeping $\lambda_{1}$ fixed at 0.2. As can be seen from Figure 13, as the weight parameters vary, the model performance within the selected parameter range shows a trend of first increasing and then decreasing. This indicates that the selected parameter range is reasonable, and that the optimal values are achieved at $\lambda_{1}=0.20$ and $\lambda_{2}=0.05$; therefore, these two values are selected as the final parameters for this paper.

(3) Ablation Comparison of Different Frequency-Domain Methods

Table 5 illustrates the impact of different frequency-domain representations on detection performance, comparing the most common FFT and DWT methods with the DTCWT method selected in this study. As the FFT method cannot directly capture low- and high-frequency information, we employed a low-pass and high-pass filter mask to extract these components from the image; however, some information loss is inevitable. As can be seen from Table 5, when FFT is used directly for frequency-domain modelling, the model’s detection accuracy decreases under various conditions. This indicates that, due to its lack of spatial locality, FFT struggles to reconstruct degraded features in local regions under complex weather conditions; naturally, the loss of information may also contribute to this issue. Following the introduction of DWT, model performance improved slightly, reaching 88.8%, 81.4%, and 82.8% under clean, hazy, and rainy conditions, respectively, with an average accuracy of 84.3%. This indicates that the Discrete Wavelet Transform can, to a certain extent, enhance the model’s local frequency-domain representation capability, although limitations remain. In contrast, the DTCWT utilised in this paper achieved the best results under all three weather conditions, as it not only preserves local information effectively but also exhibits stronger directional selectivity. The experimental results fully demonstrate that the reduced wavelet frequency-domain method adopted in this paper can effectively accomplish the object detection task in rainy and hazy weather conditions.

**Figure 14:**
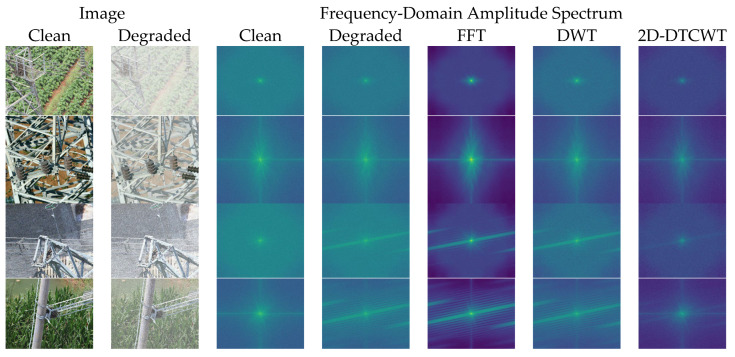
Comparison of the frequency-domain amplitudes of different frequency-domain methods.

Figure 14 displays the amplitude distribution results of some samples under different frequency-domain transformation methods, illustrating clear reference features, degraded features, and the corresponding frequency-domain amplitude responses of FFT, DWT, and 2D-DTCWT. It can be seen from Figure 14 that, for rainy or hazy images, the FFT distribution tends towards a global frequency response; however, due to poor local resolution, it struggles to capture the loss of low- and high-frequency information caused by haze and the directional interference of rain streaks. Compared to the degraded signal, the DWT shows almost no change; its limited frequency-domain discrimination capability results in no significant improvement. The frequency-domain amplitude distribution of the 2D-DTCWT exhibits greater consistency with the clear reference features. Although there remains a certain disparity in amplitude, both low-frequency and high-frequency information are preserved relatively well. More importantly, it demonstrates a stronger ability to characterise directional rain streaks; this is because the directionality of the high-frequency signals represented by the 2D-DTCWT itself is relatively complex, enabling it to better express rain streak information.

### 4.5. Computational Complexity Analysis

To assess the feasibility of our proposed method in meeting real-time requirements for UAV inspection scenarios, we compared the computational overhead of our method with that of baseline and state-of-the-art methods, specifically in terms of FLOPs, the number of parameters, and frames per second. To ensure fairness across all methods, all evaluations were conducted on an NVIDIA RTX 4090 GPU with an input resolution of 640 × 640.

As shown in Table 6, due to the introduction of an additional image-level restoration network, the method employing a Separate cascading strategy results in significant computational redundancy, with FLOPs increasing to 324.5 G and 286.2 G, whilst the inference speed drops to approximately 28–31 FPS. In contrast, as our method achieves decoupling directly within the feature domain rather than performing full-resolution image reconstruction, it significantly alleviates this computational bottleneck. Compared to the FCOS baseline method, our approach adds only 1.1 million parameters and 13.1 GFLOPs whilst still maintaining a high inference speed of 41 FPS. This result demonstrates that our method can effectively meet the real-time requirements of power line inspection tasks whilst achieving the highest mAP scores, striking an optimal balance between detection accuracy and computational efficiency.

## 5. Discussion

Table 1 and Table 2 demonstrate that the PCW-UDM algorithm proposed in this paper, when combined with the FCOS baseline model, achieves optimal detection performance on both single-class and multi-class adverse weather datasets, significantly outperforming existing Direct strategies, Separate cascade strategies, and Union optimisation algorithms such as IA-YOLO and DS-Net. Notably, the robustness analysis across different weather intensities shown in Figure 12 indicates that, as hazy concentration and rainfall intensity increase, the detection accuracy of the baseline model and other advanced methods exhibits a precipitous decline, whereas the accuracy degradation of our method is markedly smaller. This remarkable robustness stems from our model’s departure from traditional pixel-level restoration in the spatial domain; instead, it decouples degraded signals from target features within a translation-invariant complex wavelet domain, thereby avoiding semantic conflicts between image restoration and lower-level detection tasks.

From a mechanistic perspective, the performance gains of PCW-UDM arise from the synergy of its core components. Specifically:

(1) The PGLD branch effectively mitigates low-frequency contrast attenuation and energy shifts caused by atmospheric scattering through physically guided algorithms for estimating transmittance and atmospheric light.
(2) The LUHD branch implements an iterative threshold contraction method as a neural network, precisely separating directional rain streak noise from high-frequency features whilst freezing phase information to prevent geometric distortion.
(3) Ablation experiments further demonstrate that combining these two modules with a spatio-temporal cross-domain consistency loss enables the network to maintain superior detection performance in complex cross-domain scenarios.

Furthermore, in terms of visualisation results, as shown in Figure 9, Figure 10 and Figure 11, the algorithm proposed in this paper exhibits superior localisation accuracy and background interference suppression capabilities, both on synthetic datasets and on real-world RTTS adverse weather datasets. Compared to baseline models and other competing algorithms, which are prone to diffuse thermal responses, low confidence levels, and severe false positives and false negatives in complex backgrounds, PCW-UDM is able to focus attention more precisely on the target area; this powerful feature representation capability is attributed to the introduction of the two-dimensional dual-tree complex wavelet transform. Frequency-domain ablation experiments demonstrate that, compared to the FFT, which lacks spatial locality, and the DWT, which lacks translation invariance, the 2D-DTCWT is capable of both better preserving local information and exhibiting stronger directional selectivity.

Despite these advantages, we recognise that there is a trade-off between accuracy and computational complexity. Mapping spatial image features to the complex wavelet domain and applying deep decomposition algorithms inevitably increases parameter and computational overhead. However, given the vulnerability of purely data-driven models under extremely adverse weather conditions, the PCW-UDM proposed in this paper successfully achieves high-precision localisation and robust environmental adaptability under adverse weather conditions at an acceptable computational cost, providing an extremely reliable solution for foreign object detection on power transmission lines.

## 6. Conclusions

To address the issue of object feature displacement caused by severe visual degradation in UAV aerial images under adverse weather conditions, this paper proposes a novel feature-level Physics-Prior Complex Wavelet Unrolling Decoupling Module (PCW-UDM).

Based on the seamless integration of signal processing theory and deep learning, the PCW-UDM effectively overcomes the semantic conflict between image restoration and detection tasks inherent in traditional cascaded paradigms. Firstly, the method utilises the translation invariance and multi-directional selectivity of 2D-DTCWT to achieve deep decoupling of degraded features in the frequency domain. Secondly, the PGLD branch utilises a parallel, lightweight parameter estimation sub-network and an inverse atmospheric scattering model to reconstruct a clear low-frequency background, whilst the LUHD branch employs an iterative threshold contraction algorithm with deep expansion to strip away high-frequency rain streak noise in the complex polar coordinate domain which preserves the phase of the target edges. Through a specially designed spatio-temporal cross-domain consistency loss function, the model is capable of deep learning whilst maintaining alignment between the spatial semantics and frequency-domain amplitudes of the target.

Experimental results on the Multi-Adverse Dataset, which contains multiple types of foreign objects, demonstrate that the proposed method achieves mAPs of 86.5%, 83.8%, and 84.1% under clear, hazy, and rainy conditions, respectively, exhibiting superior stability across varying degradation intensities and significantly outperforming current joint optimisation methods. Furthermore, visualisation results on the real-world RTTS dataset further validate the module’s generalisation capability and false positive suppression ability.

Future research will explore the effectiveness of PCW-UDM under more severe and complex meteorological conditions. Concurrently, to address the computational overhead associated with the complex wavelet transform, we will further investigate lightweight network design strategies to meet the stringent requirements of edge computing devices and real-time inference on power transmission lines. Furthermore, we will continue to focus on collecting and processing datasets of an even higher standard, such as images of power lines captured over extended periods using fixed cameras in various weather conditions. This is because there are fundamental differences between real-world and simulated environments.

## Figures and Tables

**Table 1:** Performance comparison of the Single-Adverse Dataset.

Strategy	Method	Enhancement Module	Detection Module	Train Dataset	Clean	Hazy	Rainy	Mean
Direct	Baseline-1	-	FCOS	Clean	91.5 ± 0.2	72.8 ± 0.6	75.1 ± 0.5	79.8 ± 0.4
	Baseline-2	-	FCOS	Mixed	88.6	80.9	82.4	84.0
Separate	Sep-1	FFA-Net [57]	FCOS	Mixed	82.7	81.6	78.5	80.9
	Sep-2	MPRNet [58]	FCOS	Mixed	83.4	79.8	81.2	81.5
Union	IA-YOLO [35]	Own	YOLOv3	Mixed	87.6	83.5	84.1	85.1
	DS-Net [59]	Own	FCOS	Mixed	88.4 ± 0.3	84.8 ± 0.4	85.3 ± 0.4	86.2 ± 0.4
	**Ours**	**PCW-UDM**	**FCOS**	**Mixed**	**91.2 ± 0.1**	**88.1 ± 0.2**	**87.4 ± 0.2**	**88.9 ± 0.2**

Green indicates the best performance, blue indicates the second-best performance.

**Table 2:** Performance comparison of the Multi-Adverse Dataset.

**Strategy**	**Method**	**Enhancement Module**	**Detection Module**	**Train Dataset**	**Clean**	**Hazy**	**Rainy**	**Mean**
Direct	Baseline-1	-	FCOS	Clean	88.7 ± 0.3	61.4 ± 0.7	64.2 ± 0.8	71.4 ± 0.6
	Baseline-2	-	FCOS	Mixed	84.6	73.8	75.1	77.8
Separate	Sep-1	FFA-Net [57]	FCOS	Mixed	77.3	74.6	70.2	74.0
	Sep-2	MPRNet [58]	FCOS	Mixed	78.1	71.5	73.4	74.3
Union	IA-YOLO [35]	Own	YOLOv3	Mixed	82.0	77.8	78.4	79.4
	DS-Net [59]	Own	FCOS	Mixed	83.4 ± 0.5	79.6 ± 0.5	80.2 ± 0.4	81.1 ± 0.4
	**Ours**	**PCW-UDM**	**FCOS**	**Mixed**	**86.5 ± 0.2**	**83.8 ± 0.2**	**84.1 ± 0.3**	**84.8 ± 0.2**

Green indicates the best performance, blue indicates the second-best performance.

**Table 3:** Ablation results for different module combinations in the PCW-UDM.

Method	PGLD	LUHD	Consistency Loss	Clean	Hazy	Rainy	Mean
Baseline	✗	✗	✗	88.6	80.9	82.4	84.0
PGLD	✓	✗	✗	89.4	88.6	83.1	87.0
LUHD	✗	✓	✗	89.2	82.3	87.9	86.5
PGLD + LUHD	✓	✓	✗	90.3	86.7	86.3	87.8
Full model	✓	✓	✓	91.2	88.1	87.4	88.9

Green indicates the best performance, blue indicates the second-best performance.

**Table 4:** Analysis of the Validity of Consistency Loss.

Method	$L_{spatial}$	$L_{freq}$	Clean	Hazy	Rainy	Mean
Det loss only	✗	✗	90.3	86.7	86.3	87.8
$L_{spatial}$	✓	✗	90.7	87.5	86.7	88.3
$L_{freq}$	✗	✓	90.6	87.0	87.0	88.2
$L_{spatial}+L_{freq}$	✓	✓	91.2	88.1	87.4	88.9

Green indicates the best performance, blue indicates the second-best performance.

**Table 5:** Comparison results of different frequency-domain modelling methods.

Method	Clean	Hazy	Rainy	Mean
Baseline	88.6	80.9	82.4	84.0
+FFT	86.9	77.8	79.1	81.3
+DWT	88.8	81.4	82.8	84.3
+DTCWT (Ours)	91.2	88.1	87.4	88.9

Green indicates the best performance, blue indicates the second-best performance.

**Table 6:** Comparison of computational efficiency across different methods.

Strategy	Method	Enhancement Module	Detection Module	FLOPs (G)	Parameters (M)	FPS
Direct	Baseline-1	-	FCOS	205.3	32.1	48
Separate	Sep-1	FFA-Net [57]	FCOS	324.5	36.5	28
	Sep-2	MPRNet [58]	FCOS	286.2	52.2	31
Union	IA-YOLO [35]	Own	YOLOv3	155.4	62.3	42
	DS-Net [59]	Own	FCOS	232.6	34.5	37
	**Ours**	**PCW-UDM**	**FCOS**	**218.4**	**33.2**	**41**

## Data Availability

The original contributions presented in this study are included in the article. Further inquiries can be directed to the corresponding author.
